# Perinatal taurine exposure alters renal potassium excretion mechanisms in adult conscious rats

**DOI:** 10.1186/1423-0127-17-S1-S29

**Published:** 2010-08-24

**Authors:** Sanya Roysommuti, Pisamai Malila, Wichaporn Lerdweeraphon, Dusit Jirakulsomchok, J Michael Wyss

**Affiliations:** 1 Department of Physiology, Faculty of Medicine, Khon Kaen University, Khon Kaen 40002, Thailand; 2 Department of Cell Biology, University of Alabama at Birmingham, Birmingham, AL 35294, USA

## Abstract

Perinatal taurine exposure has long-term effects on the arterial pressure and renal function. This study tests its influence on renal potassium excretion in young adult, conscious rats. Female Sprague-Dawley rats were fed normal rat chow and given water alone (C), 3% beta-alanine in water (taurine depletion, TD)  or 3% taurine in water (taurine  supplementation, TS), either from conception until delivery (fetal period; TDF or TSF) or from delivery until  weaning  (lactation  period;  TDL  or  TSL). In Experiment 1, male offspring were fed normal rat chow and tap water, while in Experiment 2, beta-alanine and taurine were treated from conception until weaning and then female pups were fed normal rat chow and 5% glucose in drinking water (CG, TDG or TSG) or water alone (CW, TDW or TSW).  At 7-8 weeks of age, renal potassium excretion was measured at rest and after an acute saline load (5% of body weight) in conscious, restrained rats. Although all male groups displayed similar renal potassium excretion, TSF rats slightly increased fractional potassium excretion at rest but not in response to saline load, whereas TDF did the opposite. Plasma potassium concentration was only slightly altered by the diet manipulations.  In female offspring, none of the perinatal treatments significantly altered renal potassium excretion at rest or after saline load. High sugar intake slightly decreased potassium excretion at rest in TDG and TSG, but only the TDG group displayed a decreased response to saline load. The present data indicates that perinatal taurine exposure only mildly influences renal potassium excretion in adult male and female rats.

## Introduction

Perinatal taurine depletion induces low birth weight, multiple organ damage and low tissue taurine content in many organs and in plasma [1,2], but the mechanisms underlying these adverse effects remain ambiguous. Renal dysfunction with age, diabetes mellitus, hypertension, and obesity are inversely correlated with body taurine content [3]. Taurine supplementation appears to prevent some aspects of age-related renal damage, sugar-induced hypertension, ethanol-induced hypertension and drug-induced diabetes.  Perinatal taurine supplementation can prevent cardiovascular diseases in the adult offspring following maternal malnutrition [4,5] and in spontaneously hypertensive rats [6,7]. Our previous experiments indicate that perinatal taurine exposure can alter renal hemodynamics in the adult, male offspring [8]; however, the renal diuretic and natriuretic responses to an acute saline load remain within the normal control range. Taurine and potassium are major solutes inside mammalian cells, and thus, regulation of intracellular osmolarity is influenced by their relative intra- versus extra-cellular distribution [9-11]. *In vitro,* hypotonic KCl induces taurine transport out of cells by both osmotic dependent and independent mechanisms [12]. Long-term cellular volume regulation involves potassium, other ions and taurine transport across cell membrane, and this is influenced by the intracellular synthesis of taurine, and other osmolytes [13-17]. A close relation between potassium and taurine is supported by the finding that potassium depleted animals displayed decreases in plasma taurine concentration and total body taurine content [18] . Taurine is one of the major solutes that  determine interstitial fluid osmolarity in the renal medulla [19,20]. Either taurine or potassium depletion decreases taurine concentration in this area [19], thus disturbing renal urine concentration and dilution mechanisms. In addition, taurine increases renal sodium excretion [21], but it influences on renal potassium excretion is not well known.

Taurine transport across cell membrane is by sodium dependent active transport, while maintaining low sodium and high potassium levels within cells depends mainly on Na-K exchange.  Taurine, sodium and potassium imbalance appears to underline many cardiovascular diseases, especially hypertension [22,23]. High potassium intake decreases sodium pressor sensitivity and hypertension [22,24]. In contrast, low potassium diets increase sodium retention and hypertension. Low fish or taurine diets also increase risk of hypertension in the population [25,26]. The kidneys accounts for more than 90% of potassium loss, with the remainder exiting through the gastrointestinal tract. Renal potassium excretion usually reflects potassium intake and renal sodium retention. The risk of hypertension and stroke are inversely related to potassium intake in humans and animals [23,27].

In male rats, high sugar intake (since weaning) impairs renal function before either hypertension or insulin resistance development [28]. In addition, high sugar treatment increases sympathetic nerve activity in perinatal taurine depleted male rats [29]. Perinatal taurine exposure and high sugar intake also alters renal function in animals [8]. However, the effect of perinatal taurine and high sugar intake seems to be sex dependent [30]. Nevertheless, the relation between perinatal taurine exposure and renal potassium excretion in the adult offspring has not been reported. This study tests the effect of perinatal taurine on renal potassium excretion in adult conscious male and female rats, and investigates the effect of high sugar intake on the interaction.

## Materials and methods

Sprague-Dawley (SD) rats were bred from the animal unit of Faculty of Medicine, Khon Kaen University and maintained at constant humidity (60 ± 5%), temperature (24±1°C), and light cycle (0600-1800 h). All experimental procedures were preapproved by the Khon Kaen University Animal Care and Use Committee and were conducted in accordance with the National Institutes of Health guidelines.

Female SD dams were fed normal rat chow and drank water alone (Control) or water containing 3% β-alanine (taurine depletion, TD) or 3% taurine (taurine supplementation, TS) either from conception until delivery (fetal or prenatal treatment; TDF or TSF) or from delivery until weaning (lactation or postnatal treatment; TDL or TSL). In experiment 1, after weaning, the male offspring were fed normal rat chow and given water *ad libitum*. In Experiment 2, female pups were treated similarly, but from conception until weaning (perinatal treatment).  Post weaning, the female pups were treated with 5% glucose in drinking water (CG, TDG or TSG) or water alone (CW, TDW or TSW).

At 7-8 weeks of age, under thiopental sodium or Nembutal anesthesia (50 mg/kg, i.p.), all rats were implanted with femoral arterial, venous, and bladder catheters.  Forty-eight hours later, arterial pressure was continuously recorded in conscious, restrained rats, that had been acclimated to the restraint for one week (3 hours per day) prior to the experiment. Renal function was studied before, during, and after an intravenous isotonic saline infusion (a mixture of 0.5% inulin and 0.5% p-aminohippuric acid (PAH) in isotonic saline, 5% of body weight, 0.5 ml/min).

Urine and blood samples were collected before, during, and after the intravenous saline infusion.  At the end of the experiments, all animals were sacrificed and kidney (KW) and heart (HW) weights were measured.  Urine volumes was measured gravitationally, urine (U_K_) and plasma (P_K_) potassium and sodium (data not shown) by flame photometry and an automatic analyzer and urine and plasma inulin and PAH by colorimetry. Glomerular filtration rate (GFR) was estimated by inulin clearance, effective renal blood flow (ERBF) by PAH clearance and hematocrit, and renal vascular resistance was calculated by MAP/ERBF (data not shown).  Tubular potassium reabsorption was estimated from fractional potassium excretion (FE_K_), i.e., (Urine flow x U_K_)/ (GFR x P_K_).

All data were expressed as mean ± SEM and were statistically analyzed using one-way ANOVA and appropriate post hoc tests (Duncan’s Multi-Range) with a significant criterion of p < 0.05.

## Results

**Figure 1:**
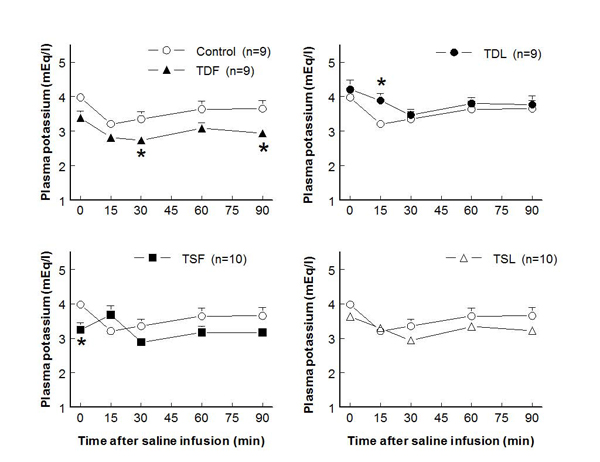
**Plasma potassium concentration before and after acute saline infusion in conscious male rats** (* P < 0.05 when compared to control of same time; see text for abbreviations)

**Figure 2:**
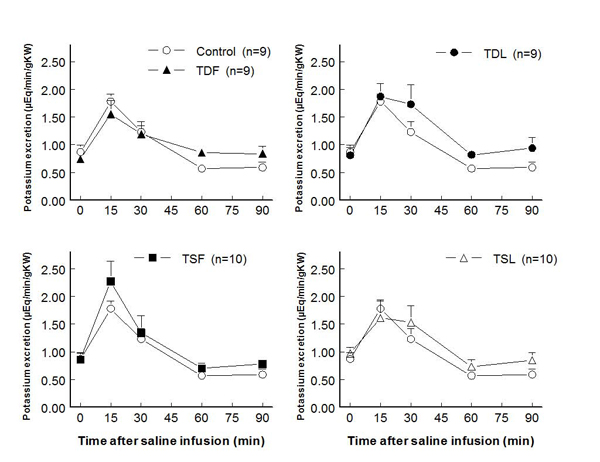
**Renal potassium excretion before and after acute saline infusion in conscious male rats** (no significant difference when compared to control of same time; see text for abbreviations)

**Figure 3:**
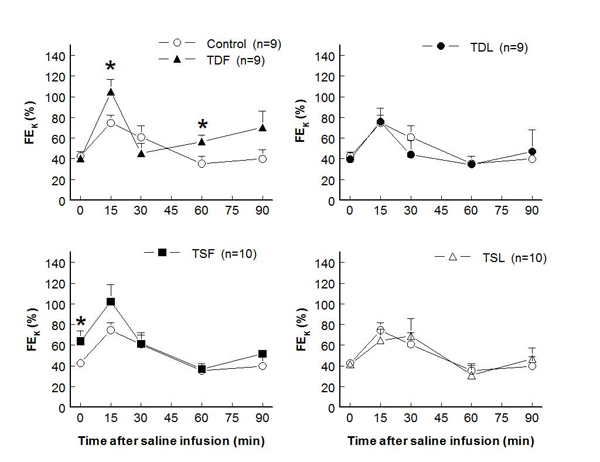
**Fractional potassium excretion (FE_K_) before and after acute saline infusion in conscious male rats** (* P < 0.05 when compared to control of same time; see text for abbreviations)

In adult male rats, plasma potassium levels at rest significantly decreased only in TDF and TSF, compared to control groups (Fig. 1).  After an acute saline load, plasma potassium slightly decreased in controls and remained at these levels throughout the study. While TDF, TDL, and TSL displayed similar patterns of plasma potassium reduction, those of TSF remained closed to their baseline level.  Renal potassium excretion was not significantly different among groups throughout the study (Fig. 2).  Compared to control, FE_K_ at rest significantly increased only in TSF while it significantly increased following the saline load only in TDF (Fig. 3).

**Figure 4:**
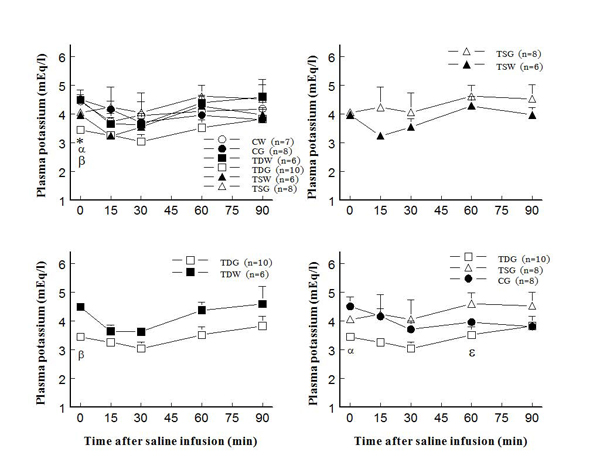
**Plasma potassium concentration before and after acute saline infusion in conscious female rats** (* P < 0.05 to CW, α to CG, β to TDW, ε to TSG; see text for abbreviations)

**Figure 5:**
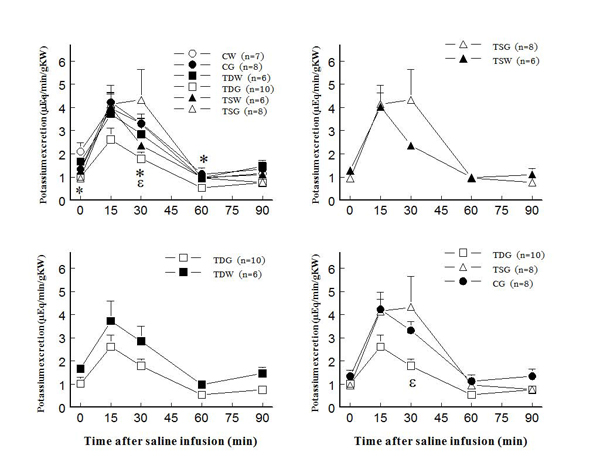
**Renal potassium excretion before and after acute saline infusion in conscious female rats** (* P < 0.05 to CW, ε to TSG; see text for abbreviations)

**Figure 6:**
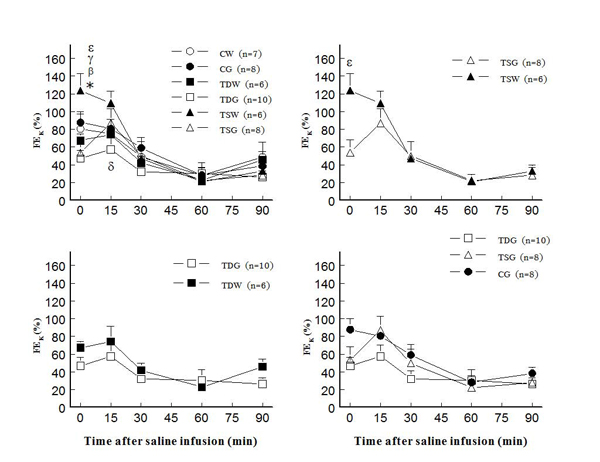
**Fractional potassium excretion (FE_K_) before and after acute saline infusion in conscious female rats** (* P < 0.05 to CW, β to TDW, γ to TDG, δ to TSW, ε to TSG; see text for abbreviations)

In adult female offspring, perinatal taurine depletion or supplementation alone did not alter plasma potassium concentration at rest or after saline load (Fig. 4).  In contrast, high sugar intake after weaning in these animals significantly decreased plasma potassium concentration in TD both at rest and after a saline load.  The high sugar intake alone (CG) did not affect plasma potassium throughout the study. Potassium excretion at rest and after a saline load also decreased only in TDG when compared to CW, CG, and TSG (Fig. 5).  In contrast, FE_K_ increased at rest only in TSW (123.5±19.0 %) when compared to other groups including TSG (53.7±14.3 %) and CW (80.5±16.4 %); however, the responses to an acute saline load did not significantly differ among groups throughout the study (Fig. 6).

## Discussion

Perinatal taurine exposure influences renal function, autonomic nervous system activity, and arterial blood pressure in mature animals [8,29,30]. In male rats, renal hemodynamics rather than sodium and water excretion mechanisms are affected by over or under exposure to taurine at early life [31], while in the female  rats, perinatal taurine depletion or supplementation alters many parameters of renal function [8]. The present study indicates that, prenatal and postnatal taurine depletion or supplementation have relatively little influence on potassium excretion in the adult male rats, although renal tubular potassium reabsorption is decreased by both, as indicated by increased FE_K_ only in prenatal depletion/supplementation.  In female offspring, perinatal taurine over  or under exposure without high sugar intake has minimal effect on renal potassium handling and plasma potassium levels, but it increases FE_K_ at rest in TSW rats. In contrast, high sugar intake decreased plasma potassium and potassium excretion in perinatal taurine depleted but not supplemented rats and returned FE_K_ of the TSW to control levels.  These changes differ from sodium and water excretion previously reported [8].

In rats, the number of nephrons is determined before birth, but their maturation continues postnatally including renal water, sodium, and potassium transports [32,33].  The present data indicate that renal potassium handling mechanisms at prenatal life are essentially dependent on optimal taurine supply by maternal nutrition.  This may reflect the fact that cell production and differentiation need intracellular potassium and taurine to maintain cell osmolarity and volume [14,16,17,32,33].  The cell volume regulation is not only necessary for cell survival against variable internal environment (extracellular fluid), but also for proper genetic and phenotypic expression of the organism [32-35].  In addition, potassium depletion and taurine deficiency are commonly and simultaneously observed in animal models [18,19].

Renal potassium excretion depends mainly on plasma potassium, glomerular filtration, and tubular reabsorption [34].  Since plasma potassium concentrations decreased both in TDF and TSF and their glomerular filtration rate, water and sodium excretion [31] were almost similar.  Thus, decreased renal tubular potassium reabsorption is the only mechanism that helps to maintain normal potassium excretion.  Although prenatal taurine over and under exposure decreased tubular potassium reabsorption, that supplementation influences only resting while that depletion only during diuretic and natriuretic responses.  Renal tubular potassium secretion increases in case of increased renal tubular luminal flow, a urine flow dependent effect [36,37].  Thus, sufficient taurine exposure is likely essential to the development of urine flow dependent potassium transport at prenatal life while its over-exposure alters that of urine flow independent potassium transport mechanisms (resting tubular transport).

The long-term effect of perinatal taurine exposure on the arterial pressure control and its high sugar intake response is sex dependent [30].  The present experiment further indicates that its effect on renal function also differs between male and female rats.  Perinatal taurine supplementation increased FE_K_ only at rest in the female similarly to that in the male rats.  In contrast to the male, perinatal taurine depletion without high sugar intake had no any effect in the female rats both at rest and after a saline load.  Nevertheless, tubular potassium transport rather than glomerular filtration is most adaptive to perinatal taurine exposure in both sexes.  Glomerular filtration rates but not plasma potassium levels at rest slightly decreased only in the TSW [8].  A decrease in tubular potassium reabsorption simultaneously with a decrease in its filtered load may be the typical adaptation of the kidney to maintain potassium balance in TSW animals.  Under normal situation, potassium excretion needs to be matched with a daily potassium input especially from diets [34,35].

High sugar intake, plasma insulin levels, insulin resistance, and sympathetic nerve activity are implicated in body potassium balance [38,39].  Hyperinsulinemia and increased sympathetic nerve activity decreased plasma potassium levels and increased renal tubular potassium reabsorption.  Our previous experiments indicate that perinatal taurine depletion or supplementation with or without the high sugar intake does not induce insulin resistance and hyperglycemia in both male and female SD rats [29,30].  In the present study, the perinatal taurine depletion treated with a high sugar diet decreases renal potassium excretion likely due to decreased plasma potassium.  Normal FE_K_ between TDW, TDG, and CG groups indicates their normal tubular potassium reabsorption.  The fact that the high sugar intake since weaning restored FE_K_ of TS rats to CG and CW groups, suggesting an interaction between a high sugar diet and perinatal taurine exposure to alter renal function in adult life [8].

## Conclusion

Taurine and potassium are main solutes inside all mammalian cells. Positive potassium balance and high taurine content are observed in perinatal life.  The present study suggests that prenatal rather than postnatal taurine exposure is critical to adult renal potassium transports in both male and female rats.  These changes may be modified by high sugar intake at later life.  Molecular mechanisms have to be further studied.

## List of abbreviations used

CW: control with water intake alone; CG: control with high sugar intake; TD: taurine depletion; TDF: prenatal taurine depletion; TDL: postnatal taurine depletion; TDW: perinatal taurine depletion with water intake alone; TDG: perinatal taurine depletion with high sugar intake; TS: taurine supplementation; TSF: prenatal taurine supplementation; TSL: postnatal taurine supplementation; TSW: perinatal taurine supplementation with water intake alone; TSG: perinatal taurine supplementation with high sugar intake; BW: body weight; HW: heart weight; KW: kidney weight; SD: Sprague Dawley; i.p.: intraperitoneal; SEM: standard error of means; P_K_: plasma potassium concentration; U_K_: urine potassium concentration; PAH: p-aminohippuric acid; GFR: glomerular filtration rate; ERBF: effective renal blood flow; MAP: mean arterial pressure; FE_K_: fractional potassium excretion

## Competing interests

The authors declare that they have no competing interests

## Authors’ contributions

Sanya Roysommuti: research proposal design, data analysis, article preparation, correspondence. Pisamai Malila: research proposal preparation, data collection and analysis. Wichaporn Lerdweeraphon: research proposal preparation, data collection and analysis. Dusit Jirakulsomchok: research consult, article preparation. J Michael Wyss: research consult, article preparation.
